# Progress in vaccination towards hepatitis B control and elimination in the Region of the Americas

**DOI:** 10.1186/s12889-017-4227-6

**Published:** 2017-04-17

**Authors:** Alba Maria Ropero Álvarez, Silvia Pérez-Vilar, Carmelita Pacis-Tirso, Marcela Contreras, Nathalie El Omeiri, Cuauhtémoc Ruiz-Matus, Martha Velandia-González

**Affiliations:** 1 Unit of Comprehensive Family Immunization. Department of Family, Gender and Life Course, Pan American Health Organization (PAHO/WHO), 525 23rd St. Nw, Washington DC, 20037 USA; 2 Current address: U.S. Food and Drug Administration, 10903 New Hampshire Ave, Silver Spring, MD 20993 USA

**Keywords:** Hepatitis B vaccine, Hepatitis B elimination, Birth dose, Region of the Americas, Mother-to-child-transmission

## Abstract

**Background:**

Over recent decades, the Region of the Americas has made significant progress towards hepatitis B elimination. We summarize the countries/territories’ efforts in introducing and implementing hepatitis B (HB) vaccination and in evaluating its impact on HB virus seroprevalence.

**Methods:**

We collected information about HB vaccination schedules, coverage estimates, and year of vaccine introduction from countries/territories reporting to the Pan American Health Organization/World Health Organization (PAHO/WHO) through the WHO/UNICEF Joint Reporting Form on Immunization. We obtained additional information regarding countries/territories vaccination recommendations and strategies through communications with Expanded Program on Immunization (EPI) managers and national immunization survey reports. We identified vaccine impact studies conducted and published in the Americas.

**Results:**

As of October 2016, all 51 countries/territories have included infant HB vaccination in their official immunization schedule. Twenty countries, whose populations represent over 90% of the Region’s births, have included nationwide newborn HB vaccination. We estimated at 89% and 75%, the regional three-dose series and the birth dose HB vaccination coverage, respectively, for 2015. The impact evaluations of infant HB immunization programs in the Region have shown substantial reductions in HB surface antigen (HBsAg) seroprevalence.

**Conclusion:**

The achievements of vaccination programs in the Americas suggest that the elimination of perinatal and early childhood HB transmission could be feasible in the short-term. Moreover, the data gathered indicate that the Region may have already achieved the 2020 WHO goal for HB control.

## Background

Hepatitis B (HB) is a liver infection caused by the Hepatitis B virus (HBV), transmitted by percutaneous or mucosal exposure to blood or body fluids of an infected person. Despite availability of effective vaccines and antiviral treatments, HBV infection continues to be a significant cause of disease burden and mortality worldwide [1].

Preventing HBV infection through vaccination has proven to be the most effective measure to reduce complications, decrease the reservoir of persons with HB chronic infections, and eliminate HBV transmission [2]. The risk of developing a chronic infection is highest for infants infected at birth or prior to six months of age. HB vaccine is, therefore, given at birth and is followed by two or three doses in order to prevent perinatally-acquired chronic infection and early childhood HB virus transmission [3]. HB vaccination is also recommended for adults in high-risk groups including healthcare workers (HCWs), sex workers, men who have sex with men, transgender persons, prison inmates, injection drug users, indigenous population, hemodialysis/transplant patients, people living with HIV, household contacts, and pregnant women at risk [1].

The World Health Organization (WHO) established a target for introducing HB vaccination into national immunization programs by 1995 for countries with an HBV carrier rate of ≥8%, by 1997 for all countries [4], and advocated for the administration of a birth dose to all newborns within 24 h from birth by 2009 [1]. WHO also established the goals of reaching 90% vaccination coverage for a third vaccine dose among infants and 50% for the birth vaccine dose by 2020, and set a target for global elimination of HBV infection as a major public health threat for 2030 [5]. Prevention of mother-to-child transmission (MTCT) through timely HBV birth dose vaccination, universal infant vaccination, and vaccination of high-risk groups, together with optimal HB diagnosis and treatment, have been identified as crucial strategies for HBV elimination.

**Fig. 1:**
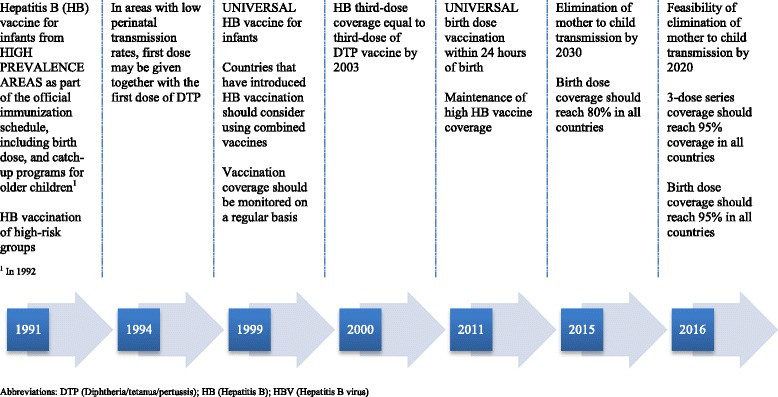
Evolution of hepatitis B vaccination recommendations in the Americas

HB vaccination was gradually implemented in the Americas starting in 1982 [6]. The Pan American Health Organization/World Health Organization’s (PAHO/WHO) Technical Advisory Group on Vaccine-preventable Diseases (TAG), that provides advice, reviews progress of national immunization programs, and promotes regional goals and strategies for immunization, endorsed and, subsequently adapted, WHO recommendations for HB control and elimination to the specificities of the Region (Fig. 1) [7]. PAHO’s Hepatitis Technical Advisory Committee and PAHO’s Core Group on Hepatitis were established in 2015 to support the elimination of viral hepatitis as a public health threat, including elimination of MTCT and early childhood transmission, considered milestones on the road to HBV infection elimination. Currently, PAHO/WHO regional plans of action aim to facilitate the integration of vaccination programs and hepatitis programs at the country/territory level [5, 8, 9].

We aimed to summarize progress to date in countries/territories of the Americas in introducing and implementing HB vaccination, as well as in evaluating its impact on HBV seroprevalence.

## Methods

We collected information on HB vaccination schedules and coverage estimates among newborns and infants; and year of vaccine introduction by countries/territories’, using the WHO/UNICEF Joint Reporting Forms on Immunization sent by the countries to the Pan American Health Organization/World Health Organization (PAHO/WHO) [10]. We used U.S. National Immunization Surveys to obtain coverage data from the US and Puerto Rico [11]. We confirmed information on vaccination schedules and year of introduction through communications with national EPI managers, PAHO/WHO focal points, and official ministries of health’ websites. We identified and reviewed vaccine impact studies conducted in Latin America. We also reviewed relevant impact studies conducted in the US and Canada, and published vaccination coverage estimates among high-risk groups to provide a comprehensive picture of vaccination efforts in the whole region.

To estimate regional HB vaccination coverage, we used data from the last five-year period available (2010–2015), considering that regional verification processes require at least five years of high HB vaccination coverage [12]. Coverage estimates exceeding 100% were considered 100% in our analyses. As recommended by WHO, we used linear interpolation to estimate missing coverage values, provided that two or more values from other years were available [13]. If no coverage data were available for the last year included in the report, we used the estimate reported in the prior year [13]. If a coverage estimate was available for only one of the years in the analysis period, the country was excluded from the regional coverage estimates for the missing years. Countries that have not yet adopted HB vaccination policies were assumed to have 0% coverage [12]. For newborns and infants, we used, as denominators, the average annual births for 2010–15, as available, from the PAHO/WHO Regional Core Health Data Initiative [14].

## Results

### Introduction of HB vaccination in the official immunization schedules

To date, all countries/territories of the Americas have included HB vaccine in their childhood immunization schedules. It was gradually implemented between 1991 and 2012, starting with the US and ending with Bonaire, Haiti, and Saba (Table 1). All countries/territories use HB-containing combination vaccines except for Canada (in six provinces, through school-based immunization programs), Mexico, and Costa Rica, which use monovalent vaccines.

**Table 1:** Hepatitis B official vaccination schedule, year of vaccine introduction, reported three-dose series vaccination coverage, year of birth dose vaccination introduction, and reported birth dose vaccination coverage by country/territory in the Americas, 2010–2015

	Three-dose series among infants ages <1 year^a^								Birth dose						
	Current vaccination schedule	Introduction	Vaccine coverage (%)						Introduction	Vaccine coverage (%)					
		(Year)	2010	2011	2012	2013	2014	2015	(Year/Status)	2010	2011	2012	2013	2014	2015
Northern America															
Bermuda	6, 7, 12 m	1997	93	90	92	91	97	85	----^g, h^	-	-	-	-	-	-
Canada	0, 2, 6 m^2^or 2, 4, 6 m	1993^b^	-	70	70	75	74^f^	73	1983^g^, 1993^b^	-	-	-	-	-	-
United States^a^	0, 1–2, 6–18 m	1991	92	91	90	91	92	92^f^	1991	56	60	63	65	64	64^e^
Mexico	0, 2, 6 m	1999	93	98	99	79	84	82	2007	84	98	94^e^	89	94^e^	98
Central America															
Belize	2, 4, 6 m	1999	96	95	98	95	95	94	No	0	0	0	0	0	0
Costa Rica	0, 2, 6 m	1992^d^, 1997	89	84	91	94	91	92	1988^g^, 1997	86	88^e^	90	90^e^	89	89^e^
El Salvador	2, 4, 6 m	1999	89	89	92	92	93	91	2015	-	-	-	-	-	-
Guatemala	0, 2, 4, 6 m	2005	94	88	96	93	73	74	2010	15	30	35	37	22	32
Honduras	0, 2, 4, 6 m	2000	100^e^	100^e^	88	87	85	85	2007	99	98	98	100	100	72
Nicaragua	2, 4, 6 m	1999	100^e^	100^e^	100^e^	100^e^	100^e^	100^e^	No	0	0	0	0	0	0
Panama	0, 2, 4, 6 m	1999	94	87	85	80	80	73	2002	89	93	87	79	84	85
Andean area															
Bolivia	2, 4, 6 m	2000	80	82	80	81	85	89	No	0	0	0	0	0	0
Colombia	0, 2, 4, 6 m	1992^d^, 1994	88	85	92	91	90	91	1994^d^, 2001	74	77	85	82	86	87
Ecuador	0, 2, 4, 6 m	1999	100^e^	100^e^	100^e^	87	83	78	2005^d^, 2009^i^	5	7	16	69	79	75
Peru	0, 2, 4, 6 m	1991^c^, 1996^d^, 2003	93	91	95	88	88	90	1996^c^, 2003	74	76	81	82	78	79
Venezuela	0, 2, 4, 6 m	2000	78	78	81	82	78	87	2008	73	78	67	80	82	89
Southern cone and Brazil															
Argentina	0, 2, 4, 6 m	2000	94	93	91	94	94	94	2000	82	85	88	85	87	84
Brazil^a^	0, 2, 4, 6 m	1989^c^; 1991^d^, 1998	96	98	96	100^e^	96	96	1998	-	-	-	39	88	91
Chile	2, 4, 6 m	2005	92	94	90	90	95	96	----^g^	-	-	-	-	-	-
Paraguay	2, 4, 6 m	2002	76	76	74	73	74	80	----^g^	-	-	-	-	-	-
Uruguay	2, 4, 6, 15 m	1999	95	95	95	95	95	95	1991^g^	-	-	-	-	-	-
Latin-Caribbean															
Cuba	0, 2, 4, 6 m	1990	96	100^e^	100^e^	96	100^e^	100^e^	1992	99	99	99	100	99	99
Dominican Republic	0, 2, 4, 6 m	1994	83	80	74	80	89	81	1997	80	82	74	78	82	79
French Guiana	0, 2 11 m	1994	-	-	-	-	-	-	2008	-	-	-	-	-	-
Guadeloupe	2, 4, 11 m	----	-	-	-	-	-	-	----^g^	-	-	-	-	-	-
Haiti	6w 10w 14w	2012	0	0	0	85	60	72	No	0	0	0	0	0	0
Martinique	2, 4, 11 m	-----	-	-	-	-	-	-	----^g^	-	-	-	-	-	-
Puerto Rico^a^	0, 1–2, 6–18 m	1994	-	-	-	-	93	-	1999	-	-	-	-	77	-
Non Latin-Caribbean															
Anguilla	2, 4, 6 m	1997	100^e^	100	100^e^	100	100^e^	100^e^	No	0	0	0	0	0	0
Antigua and Barbuda	2, 4, 6 m	2000	98	99	98	98	100^e^	100^e^	No	0	0	0	0	0	0
Aruba	1, 3, 9 m	2003	96	95^f^	94^f^	94^f^	93^f^	92	No	0	0	0	0	0	0
Bahamas, The	2, 4, 6 m	2001	98	95	96	97	96	95	No	0	0	0	0	0	0
Barbados	2, 4, 6 m	2001	86	91	87	91	94	97	No	0	0	0	0	0	0
Bonaire	2 m, 14w, 5 m, 11 m	2012	-	-	-	-	-	-	No	0	0	0	0	0	0
Cayman Islands	0, 6w, 9 m	1997	75	75	94	86	88	87	1997^j^	-	-	-	-	87	85
Curaçao	2, 4, 6 m	2011	0	0	-	95	-	-	No	0	0	0	0	0	0
Dominica	2, 4, 6 m	2006	98	98	97	97	97	98	----^g^	-	-	-	-	-	-
Grenada	6-8w 16-20w 24-28w	2001	97	95	97	100^e^	97	92	----^g^	-	-	-	-	-	-
Guyana	2, 4, 6 m	2001	95	93	97	98	98	95	----^g^	-	-	-	-	-	-
Jamaica	6w 3 m 5–6 m	2003	94	92	96	93	92	91	No	0	0	0	0	0	0
Montserrat	2, 4, 6 m	1999	100^e^	100	94	97^f^	100	100	----^g^	-	-	-	-	-	-
Saba	2, 3, 4, 11 m	2012	0	0	100	-	-	-	----^g^	-	-	-	-	-	-
St. Kitts & Nevis	0, 2, 4, 6 m	1999	96	98	100^e^	97	98	94	2015	0	0	0	0	0	100
St. Lucia	3, 5, 7 m	2002	97	100	98	100^e^	99	100^e^	----^g^	-	-	-	-	-	-
St. Vincent & Grenadines	2, 4, 6 m	2003	100^e^	96	96	100^e^	98	100^e^	----^g^	-	-	-	-	-	-
Sint Eustatius	2, 3, 4, 11 m	1997	-	-	-	-	-	-	No	0	0	0	0	0	0
Sint Maarten	2, 3, 6 m	2000	90	92	91	92	95^f^	97	No	0	0	0	0	0	0
Suriname	2, 4, 6 m	2003^d^; 2005	86	86	84	86	85	89	2005	0	0	51	45	64	65
Trinidad & Tobago	3, 4–5, 6 m	2003	90	90	92	92	92	90	No	0	0	0	0	0	0
Turks & Caicos Islands	2, 4, 6 m	1999	95	87	95	100^e^	91	94	----^g^	-	-	-	-	-	-
Virgin Islands (UK)	2, 4, 6 m	1999	87	92	97	82	80	97	No	0	0	0	0	0	0

^a^Vaccination coverage data for the US and Puerto Rico were obtained from the National Immunization Survey (NIS) among children aged 19–35 months [11]. Birth dose coverage data for Brazil was obtained from the National Immunization Programme, Ministry of Health

^b^Each province in Canada decides which vaccination policy implements within the NACI recommendations (National Advisory Committee on Immunization). Alberta, Saskatchewan, Manitoba, Ontario, Nova Scotia, Newfoundland and Labrador maintain a school-based immunization programme. Currently, only New Brunswick, Northwest Territories, and Nunavut are administering a dose at birth

^c^Pilot studies/vaccination campaigns

^d^Year of introduction in risk/selected areas

^e^The country/territory reported more than 100% coverage

^f^Linear interpolation was used to estimate missing coverage values that two or more values from other years were available. If no coverage data were available for the last year included in this report, the estimate remained the same as the previous year [13]

^g^Only infants born to HBsAg positive mothers or mothers having acute hepatitis during pregnancy

^h^HB vaccine in was routinely administered at birth in and around 1998

^i^The dose was given within 1 month of age until 2013. In 2016, the information systems were changed in order to recognize vaccination within 24 h

^j^The dose is given 48 h from birth

*Abbreviation*: *NA* (non-applicable)

As of October, 2016, 35 (69%) of 51 countries/territories have included the birth HB vaccine dose into their immunization schedules: 20 countries/territories implemented it nationwide, and 14 countries/territories restricted its use to infants born to HBsAg-positive mothers. While Canada health authorities recommend universal birth dose vaccination, this, to date, has only been implemented in three provinces (Table 1).

Thus, the vast majority of countries/territories in the Americas use combination vaccines and administer them at 2, 4 and 6 months, with an additional dose at birth among countries with the corresponding policy in place.

In addition, at least six countries, including but not limited to Argentina, Brazil, Cuba, Peru, Uruguay and the US, have carried out catch-up vaccination and expanded vaccination to older age groups as complementary HB control strategies.

### HB vaccination coverage among newborns and infants

**Fig. 2:**
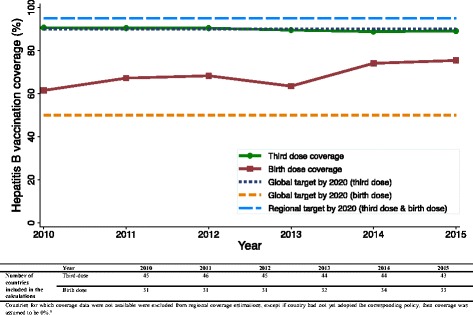
Reported hepatitis B vaccination coverage for the three-dose series among children aged less than one year and for the birth dose among newborns, The Americas, 2010–2015

For the period 2010–15, the average vaccination coverage for the three-dose series of HB vaccine among countries/territories of the Region ranged from 36% in Haiti, which introduced HB vaccination in 2012, to 99% in Nicaragua, Anguilla, and St Lucia. Of 42 countries/territories for which coverage data were available for the entire period, 31 (74%) reported a vaccination coverage ≥90%. We estimated at 89% the regional three-dose series vaccination coverage for 2015. Figure 2 shows regional vaccination coverage estimates per year for 2010–15.

For the same period, the birth dose vaccination coverage ranged from 17% in St Kitts and Nevis, which only introduced birth dose vaccination in 2015, to 99% in Cuba. For 2015, we estimated the regional coverage for the birth dose at 75%, including all countries, and at 80% when restricting to countries with universal birth dose vaccination policies (Fig. 2).

### Targeting high-risk groups for HB vaccination

In 1991, the US required employers to ensure that the HB vaccine series was made available at no cost to all employees who have occupational exposure. Subsequently, third-dose vaccine coverage increased up to 67% and incidence among HCWs declined strikingly (95%). This reduction was 1·5-fold greater than the reduction in incidence in the general US population during the same period [15]. Despite early recommendations to vaccinate HCWs [6], studies from Mexico showed low vaccination coverage in 2003 and 2010, especially for the full vaccination series (6–19%) [16, 17]. Brazil, which has vaccinated HCWs since 1993, reported, in 2005, 2006 and 2010, full series coverage ranging between 53% and 76% [18–20]. Low rates of HBsAg positivity were found in seroprevalence studies conducted among HCWs in the US (0·1%) and Brazil (0·8%) [21]. Peru was the first country to start work towards achieving full HB vaccine coverage for HCWs under the Global Plan of Action on Workers’ Health, set by the 2007 World Health Assembly [22]. During the first campaign, 96% of workers received the first dose, and 54% received the third-dose during the same year [23].

Regarding vaccination among other high-risk groups, 57% vaccination coverage among HIV-infected patients was assessed in South Brazil during 2012–13 [24]. Serological profiles among crack cocaine users in Central Brazil suggested a lower HB vaccination coverage (18%) [25]. Given difficulties in identifying high-risk groups, Argentina, Brazil, and Cuba have expanded their HB vaccination policies to cover the entire population regardless of age or risk profile.

In 2011, the US recommended HB vaccination for unvaccinated adults ages 19–59 years with diabetes; vaccination of unvaccinated adults aged ≥60 years with diabetes was left to the discretion of the treating clinician after assessing their risk for HB infection and the likelihood that they will have an adequate immune response to vaccination. HB vaccination coverage in 2014 among this risk group showed no improvement over estimates obtained before the recommendation [26].

### Countries/territories efforts to evaluate the impact of HB vaccination

The evaluation of the impact of infant HB immunization programs in the Region has shown significant reductions in HBsAg prevalence. Two studies from Peru reported a substantial reduction in HBV infection, 83% and 92%, respectively, among children ages <5 years living in high-prevalence areas. In these areas, HB vaccine was included in the immunization schedule as a birth dose followed by two or three doses, and catch-up vaccination of older children was carried out [27, 28]. Brazil, formerly an intermediate-high-endemicity country, has observed an important reduction in endemicity levels as a result of national control strategies including immunization of infants, adolescents, and adults ≤49 years of age [29, 30]. In Colombia, following eight years of HB vaccination, the prevalence of HBV infection and carriage decreased by 60%, to 75% among children ages 1–12 years in former highly endemic areas [31]. A study conducted among native Alaskan children, a population with high infection prevalence, reported the elimination of transmission of chronic HBV infection following the introduction of a comprehensive control program that included timely birth dose administration, routine infant vaccination, and catch-up vaccination of older children and adults [32]. Similar results were observed in Hawaii, which, additionally, implemented vaccination as immigration and school entry requirement, [33] and in areas of the Peruvian Amazon, where indigenous children were vaccinated with three-dose series (≥60% coverage) and a timely-administered birth dose (≥40% coverage) [28].

In the US, which implemented a comprehensive vaccination program including universal vaccination of infants, screening of all pregnant women with post-exposure prophylaxis, catch-up vaccination of adolescents, and vaccination of high-risk adults, a reduction of 68% in HBV infection prevalence among children was observed, but infection rates changed little among adults [34]. In Bolivia, another country with low endemicity prior to introduction of universal vaccination among children aged <1 year, a serological study with limited sample size (424 individuals) identified a persistent low prevalence of HBV infection after a decade of universal vaccination among children ages 5–16 years [35]. Quebec and British Columbia, like other provinces of Canada, initially opted for school-based pre-adolescent or adolescent HB immunization programs instead of routine infant vaccination. Two decades after starting this policy, along with vaccination of high risks individuals and routine pre-natal HBsAg screening, Quebec, a low endemicity region, showed a significant reduction in the reported incidence of acute HB cases (97%, *p* < 0·001) not only in adolescents, but also in children and adults aged 20–29 years. It also observed a decrease in the rate of newly reported chronic HB of 66% (*p* < ·0001) [36]. British Columbia, experienced historic declines in acute HB infection rates in the general population, and eliminated acute HB infection among the immunized adolescent cohorts upon ten years of program implementation [37].

## Discussion

**Fig. 3:**
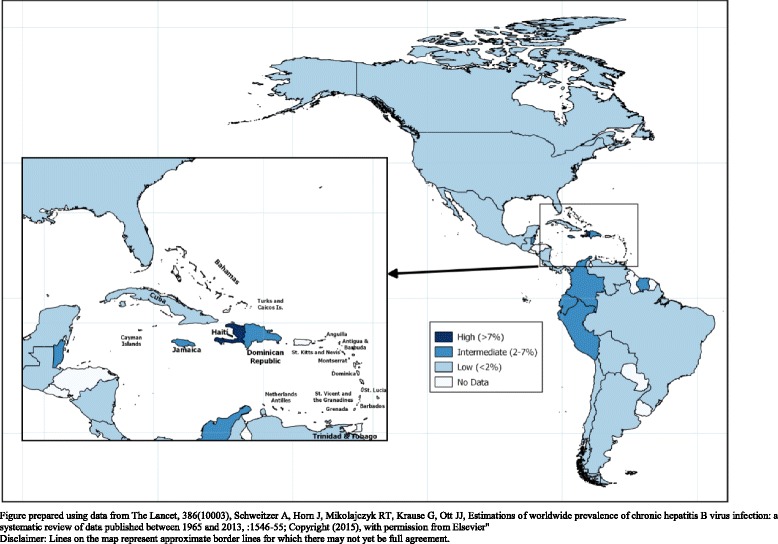
Estimated Hepatitis B surface antigen seroprevalence in the Americas, 1957–2013 [41]

Significant progress has been made in the Americas since HB vaccines were introduced three decades ago. Based on administrative coverage data, the Region may have already achieved the targeted objective set up by WHO of reaching 90% third-dose coverage among infants, and 50% birth dose vaccine coverage by 2020 [5]. The prevalence of chronic HB infection has decreased significantly, particularly in areas formerly classified as highly endemic [38–40]. Data from a recent literature review suggest that regional HBsAg seroprevalence is below 1% and that only some areas maintain HBsAg seroprevalence estimates ≥2% (Fig. 3) [41]. To date, all countries/territories have included childhood HB vaccination in their immunization schedules, 20 countries, over 90% of the Region’s birth cohort, have included universal newborn vaccination, and multiple countries have used catch-up vaccination of older individuals and high-risk groups. The observed reduction in HBsAg seroprevalence is consistent with that reported by other regions where routine infant immunization programs with high vaccination coverage were also implemented. [38, 42, 43].

In 2016, PAHO/WHO’s TAG assessed the feasibility of eliminating mother-to-child transmission (MTCT) and early childhood transmission from all countries in the Americas by 2020, defined as reaching HBsAg seroprevalence of ≤0·1% among children ages less than 5 years. The committee concluded that both eliminations would be feasible by ensuring 95% coverage for the third-dose among infants ages <1 year, and 95% coverage of timely birth dose vaccination [7].

Achievements to date are promising, but challenges remain. Although, under current policies, vaccination covers over 90% of the Region’s births, thirty countries/territories, especially from the Caribbean sub-region, have yet to include universal HB birth dose into their vaccination schedules [14], Moreover, 14 of these countries/territories administer a birth dose only to infants born to HBsAg positive mothers, but recommend screening of pregnant women. Although the efficacy of protecting newborns from chronic HBsAg carrier status with passive-active immunoprophylaxis is >90%, it may not be feasible, in practice, to screen all pregnant women or to include HB immunoglobulin for prophylaxis in low and middle-income countries. Additionally, this strategy fails to provide early pre-exposure protection to babies born to uninfected women who may live with infected household contacts [44]. Thus, PAHO/WHO recommends the universal administration of the birth dose within 24 h of birth in all countries/territories of the Region, even those with HB low endemicity [1, 7].

Further efforts are warranted to reach high uptake of the three-dose series and boost birth dose administration in all countries. Although only approximately 27% of the municipalities reported a third dose vaccination coverage <80% in 2015 (PAHO/WHO unpublished data), data reported may not reflect actual HB coverage among children living in underserved areas and/or pertaining to sub-populations at higher risk [45]. Data from a household survey carried out in the Peruvian amazon in 2012 revealed that birth dose coverage among indigenous and non-indigenous infants ages <6 months of age were 66% and 83%, respectively [46]. Given that regional data from 2014 indicated that 94% of births took place in hospitals (92% in Latin America/Caribbean) [47], birth dose administration policies that include a pre-defined time period for vaccine administration prior to discharge from health facilities might facilitate achieving higher timely coverage across the Region [12]. Thus, making HB vaccine available in maternity hospitals and delivery rooms, to be administered simultaneously with Vitamin K injection/BCG vaccine for instance, is highly recommended. Data also showed that 96% of deliveries (94% in Latin America/Caribbean) were attended by skilled health personnel [47]. Nonetheless, there are still countries, such as Haiti and Guatemala, in which the percentage of births attended by skilled personnel remains low [47], and areas in which inequalities may remain concealed within the aggregated data. Data from a household survey carried out in the Peruvian amazon in 2012 showed that 14% and 61% of indigenous and non-indigenous women, respectively, delivered their babies at heath facilities [46]. Thus, immunization programs should be integrated with maternal and neonatal services, and should facilitate training on vaccine handling, administration and reporting of birth dose vaccination. Recent data from high-endemic Colombian amazon areas showed that although 79% of the children ages 6 months-8 years received a monovalent dose of HB vaccine, only 31% were vaccinated within the first 24 h from birth [48]. Health services might consider organizing reach out activities for women delivering at home, offering vaccine to newborns. Birth dose coverage should be recorded separately for timely birth dose and for birth dose given after 24 h, thus, facilitating detection of problems with timely vaccination. Current practices and information systems should be adapted for that purpose.

The assessment of the elimination of perinatal and early childhood transmission of HB in the Americas is a lengthy process requiring input from various data sources. Seroprevalence surveys are a crucial step in the elimination verification, and should be carried out country by country. Countries may benefit from conducting operational research that would help focus the final efforts towards elimination.

While universal infant vaccination has reached clear priority among countries/territories, strategies to control transmission among high-risk groups will also be key in the elimination process. As vaccination coverage among high-risk groups including HCWs is not systematically assessed or reported to health authorities, additional strategies need to be put in place to document vaccine uptake in these populations. Also, catch-up vaccination targeting cohorts of children and adolescents born before routine HB introduction or before high vaccine coverage was achieved will also be needed in some countries [1, 7].

## Conclusions

The achievements of vaccination programs against HB in the Americas to date suggest that the elimination of perinatal and early childhood transmission could be feasible in the short-term, as recently assessed by PAHO/WHO’s TAG. Moreover, the Region may have already achieved the goal of HB control as defined by WHO and set for 2020 [5]. Besides the inclusion of universal HB birth dose into all national programs, and boosting of coverage for timely birth dose and infant HB vaccination, specially among indigenous and other populations living in underserved areas, vaccination efforts among older susceptible individuals in certain countries and high-risk groups, including HCWs, injectable drug users and HIV-infected people, should be implemented to help reach not only early childhood and MTCT transmission, but also the elimination of HB as a major public health problem.

## Abbreviations

DTP: Diphtheria/tetanus/pertussis
EPI: Expanded Program on Immunization
HB: Hepatitis B
HBsAg: Hepatitis B virus surface antigen
HBV: Hepatitis B virus
HCWs: Health care workers
JRF: WHO/UNICEF Joint Reporting Forms on Immunization
KAP: Knowledge, Attitude and Practices
MTCT: Mother-to-child-transmission
PAHO/WHO: Pan American Health Organization/World Health Organization
TAG: Technical Advisory Group on Vaccine-preventable Diseases
US: United States
WHO: World Health Organization
